# Efficacy and safety of repetitive transcranial magnetic stimulation for major depressive disorder in Chinese patients: A systematic review and meta-analysis

**DOI:** 10.1097/MD.0000000000044663

**Published:** 2025-09-19

**Authors:** Xianyan He, Xiaofeng Gao

**Affiliations:** a Department of Psychiatry, The Third Hospital of Quzhou, Quzhou, Zhejiang, China.

**Keywords:** intermittent theta burst stimulation, major depressive disorder, meta-analysis, randomized controlled trial, repetitive transcranial magnetic stimulation

## Abstract

**Background:**

Major depressive disorder (MDD) imposes a substantial burden in China, with many patients experiencing inadequate response to conventional therapies. Repetitive transcranial magnetic stimulation (rTMS) and its patterned variant, intermittent theta burst stimulation (iTBS), have shown promise, but comprehensive evidence in Chinese populations is limited. This systematic review and meta-analysis evaluated the efficacy and safety of rTMS protocols in Chinese adults with MDD.

**Methods:**

Systematic searches were conducted in 5 databases (PubMed, Cochrane, Embase, CNKI, and Web of Science) until February 2025. Randomized, double-blind, sham-controlled trials of transcranial magnetic stimulation (rTMS/iTBS) targeting dorsolateral prefrontal cortex in Chinese MDD patients (DSM-IV/V criteria) were included. Methodological quality was assessed using Cochrane tools. Data analysis used RevMan 5.3, Stata 15.0, and GRADEpro for result analysis.

**Results:**

Seven randomized clinical trials (430 participants) demonstrated significant reductions in Hamilton Depression Rating Scale scores with active stimulation versus sham (standardized mean difference = −1.35, 95% confidence interval [CI]: −1.92 to − 0.78, *P* < .00001). Active stimulation also significantly improved response rates (7 studies, odds ratio = 2.45, 95% CI: 1.58–3.78) and remission rates (5 studies, odds ratio = 2.68, 95% CI: 1.61–4.48). No significant increase in adverse events (including dizziness [3 studies], nausea [3 studies], and headache [7 studies]) was observed. The overall certainty of evidence for Hamilton Depression Rating Scale reduction was rated as low due to heterogeneity and risk-of-bias concerns.

**Conclusion:**

Repetitive transcranial magnetic stimulation (including high-frequency rTMS and iTBS) is effective and safe for treating MDD in Chinese patients. These findings support its wider clinical application, although further large-scale, high-quality trials are warranted.

## 1. Introduction

Major depressive disorder (MDD) involves complex pathophysiology, with inflammation playing a significant etiological role. Analyses, such as in the Netherlands Study of Depression and Anxiety, show linear increases in inflammatory markers like CRP (*P* = .026) and IL-6 (*P* = .090) across clinical stages, with changes also evident in at-risk individuals (IL-6, *P* = .050).^[1]^ Neurotransmitter dysregulation, including autonomic imbalance reflected in abnormal heart rate variability (HRV), is another key feature. HRV biofeedback improves depressive symptoms and HRV metrics in MDD patients,^[2]^ while imbalances in GABA and glutamate also contribute.

Epidemiological data highlight MDD’s burden in China. The national China Mental Health Survey (2012–2015, n = 28,140) found higher prevalence in females, the unemployed, and those separated, widowed, or divorced. Most patients experienced functional impairment, yet only 9.5% received any treatment within 12 months, with merely 0.5% receiving adequate care.^[3]^ Regional variations exist: a Shanghai hospital survey (n = 2000) reported higher rates linked to lack of support and burdens,^[4]^ while a Hebei study (n = 14,654) found associations with age 45 to 59, female sex, marriage, higher education, employment, medical conditions, and family history.^[5]^

Transcranial brain stimulation offers novel therapeutic avenues. Repetitive transcranial magnetic stimulation (rTMS) demonstrated efficacy in severe, treatment-resistant MDD patients, significantly improving depression scores with effects lasting weeks.^[6]^ Furthermore, evaluations of transcranial stimulation, such as EEG-synchronized TMS, show active stimulation significantly outperforms sham in reducing depression severity.^[7]^ Simultaneously, safety profiles are generally favorable. rTMS and other noninvasive techniques show good safety and tolerability in pediatric populations, with low dropout rates and mostly mild, self-limiting adverse events.^[8]^

Although rTMS has emerged as a leading noninvasive treatment for MDD, particularly in Chinese populations, the available evidence remains fragmented across studies with heterogeneous methodologies, stimulation parameters, and patient characteristics. Individual trials often lack sufficient power to draw definitive conclusions, especially regarding comparative efficacy or specific subgroups. At present, there is no comprehensive synthesis focused exclusively on Chinese MDD patients. Therefore, this systematic review and meta-analysis aims to rigorously integrate current evidence on the efficacy and safety of rTMS in adult Chinese patients with MDD, to inform clinical practice and guide future research.

## 2. Materials and methods

### 2.1. Database searching

Two trained researchers (H.X. and G.X.) systematically searched the China National Knowledge Infrastructure, PubMed, EMBASE, Web of Science, and the Cochrane Library from database inception to February 25, 2025. The search strategy combined Medical Subject Headings and free-text terms, using the following keywords: (“transcranial magnetic stimulation” OR “repetitive transcranial magnetic stimulation” OR “rTMS” OR “TMS”) AND (“depression” OR “depressive disorder” OR “major depressive disorder” OR “MDD” OR “unipolar depression”) AND (“randomized controlled trial” OR “randomized clinical trial” OR “RCTs”). For Chinese databases, equivalent Chinese terms were used to ensure retrieval consistency. All included studies focused on Chinese patients diagnosed with major depressive disorder.

### 2.2. Inclusion criteria

We included randomized, double-blind, sham-controlled trials investigating the efficacy of active repetitive transcranial magnetic stimulation protocols (high-frequency rTMS, low-frequency rTMS, or intermittent theta burst stimulation [iTBS]) versus sham stimulation in adults (≥18 years) diagnosed with MDD according to DSM-IV or DSM-5 criteria. All included participants were Chinese patients.

The only difference between the intervention and control groups was the application of active versus sham stimulation. Both groups were allowed to receive or not receive concomitant antidepressant medications (e.g., escitalopram or citalopram), provided such treatment was applied equally across groups. The intervention group received at least 1 full course (typically 10–20 sessions) of stimulation targeting the dorsolateral prefrontal cortex. The control group received sham stimulation using procedures that mimicked the active intervention without delivering therapeutic magnetic pulses. Primary outcomes included changes in Hamilton Depression Rating Scale (HAMD) scores, response rate (defined as a ≥ 50% reduction from baseline), and remission rate (defined as a posttreatment HAMD score ≤ 7). Secondary outcomes included the incidence of adverse events.

### 2.3. Exclusion criteria

Studies were excluded if participants had comorbid psychiatric disorders (e.g., bipolar disorder, schizophrenia), neurological diseases, a history of substance dependence, or contraindications to neuromodulation (e.g., metal implants, epilepsy); if they suffered from serious underlying physical illnesses, major organ dysfunction, comorbid malignancies, a history of brain surgery or traumatic brain injury, severe suicidal ideation, or significant cognitive impairment (e.g., MMSE < 24) or diagnosed neurocognitive disorders; if trials permitted structured psychotherapy or additional non-pharmacological or pharmacological co-interventions (e.g., cognitive behavioral therapy, acupuncture, herbal medicine, sedative agents) beyond sham procedures or antidepressants applied equally across groups; or if data were incomplete or the study design was not a randomized controlled trial (RCT) (e.g., expert opinion articles, animal experiments, narrative reviews, or case reports).

### 2.4. Quality assessment and data extraction

Two reviewers (H.X. and G.X.) independently extracted study characteristics, including the first author, year of publication, diagnostic instruments, depression rating scales, and baseline and post-intervention scores. Data extraction was cross-checked, and discrepancies were resolved through discussion or by consulting a third reviewer. When data were incomplete or unclear, attempts were made to contact the original authors; studies with insufficient data were excluded to ensure data integrity. Risk of bias was assessed using the Cochrane Collaboration Risk of Bias tool, covering random sequence generation, allocation concealment, blinding, incomplete outcome data, selective reporting, and other potential sources of bias. Each domain was rated as low, high, or unclear risk.

### 2.5. Data analysis

Data were extracted using Excel (Microsoft Corporation, Redmond) and analyzed with RevMan 5.3 (The Cochrane Collaboration, London, UK) and Stata 15.0 (StataCorp LLC, College Station). For dichotomous outcomes, risk ratios with 95% confidence intervals (CIs) were calculated; for continuous outcomes, weighted mean differences or standardized mean differences (SMDs) were used, depending on measurement consistency. A fixed-effects model was applied when heterogeneity was low (*I*² ≤ 50%); otherwise, a random-effects model was used. Sensitivity analyses were conducted to assess result robustness. When ≥ 10 studies were included, publication bias was evaluated using funnel plots and Begg/Egger tests in Stata.

## 3. Results

### 3.1. Search results and study characteristics

A total of 836 records were retrieved from electronic databases, including PubMed (n = 101), Cochrane Library (n = 251), Embase (n = 122), CNKI (n = 171), and Web of Science (n = 191). After removing 352 duplicates, 484 records remained for screening. Following the evaluation of titles and abstracts, 387 records were excluded due to irrelevance to the research topic or ineligible study design.

Subsequently, 97 full-text articles were assessed for eligibility. Of these, 87 studies were excluded for the following reasons: not RCTs (n = 23), participants not meeting the diagnostic criteria for major depressive disorder (n = 45), absence of sham control (n = 12), insufficient outcome data (n = 5), or non-depression-specific outcomes (n = 2).

**Figure 1.**
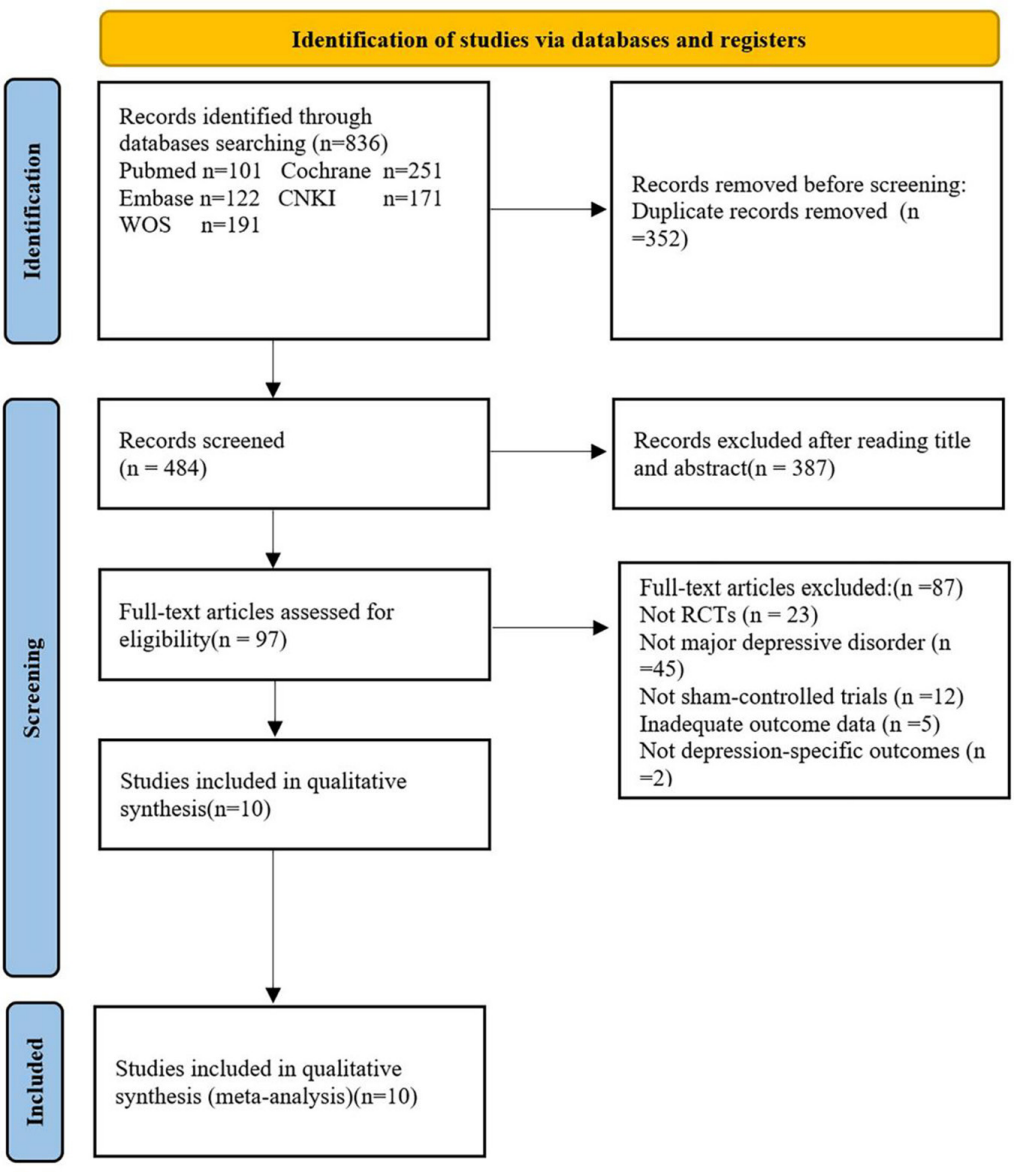
Study selection flowchart based on PRISMA guidelines.

Finally, 10 randomized, double-blind, sham-controlled trials met the inclusion criteria and were included in both qualitative and quantitative syntheses. The study selection process is detailed in Figure 1. The general characteristics of the included studies are detailed in Table 1.

**Table 1:** Principal characteristics of RCTs included in the meta-analysis.

Study (yr)	Sample size	Intervention		Background therapy	Duration (wk)	Outcome measures
	T/C	T	C			
Fu^[9]^	35/35	HF-rTMS	Sham rTMS	Duloxetine HCl	8	①④
Gu^[10]^	20/20	LF-rTMS	Sham rTMS	NR	2	②③④
Huang^[11]^	28/28	HF-rTMS	Sham rTMS	citalopram	2	①②③④
Li^[12]^	35/31	HF-rTMS	Sham rTMS	None	4	①
Li^[13]^	52/53	HF-rTMS	Sham rTMS	NR	2	①②④
Li^[14]^	28/28	HF-rTMS	Sham rTMS	Venlafaxine	2	①④
Wang^[15]^	22/21	HF-rTMS	Sham rTMS	Paroxetine	4	①②③④
Wang^[16]^	38/38	HF-rTMS	Sham rTMS	Escitalopram	NR	②③
Zhang^[17]^	29/30	iTBS	Sham iTBS:	NR	3	②③④
Zheng^[18]^	19/15	HF-rTMS	Sham rTMS	Escitalopram	4	②

C = control group, HF-rTMS = high frequency repetitive transcranial magnetic stimulation, LF-rTMS = low frequency repetitive transcranial magnetic stimulation, NR = not reported, RCTs = randomized controlled trial, T = treatment group. Outcome measures: ①: HAMD, ②: response rate, ③: remission rate, ④: adverse events.

### 3.2. Risk of bias

**Figure 2.**
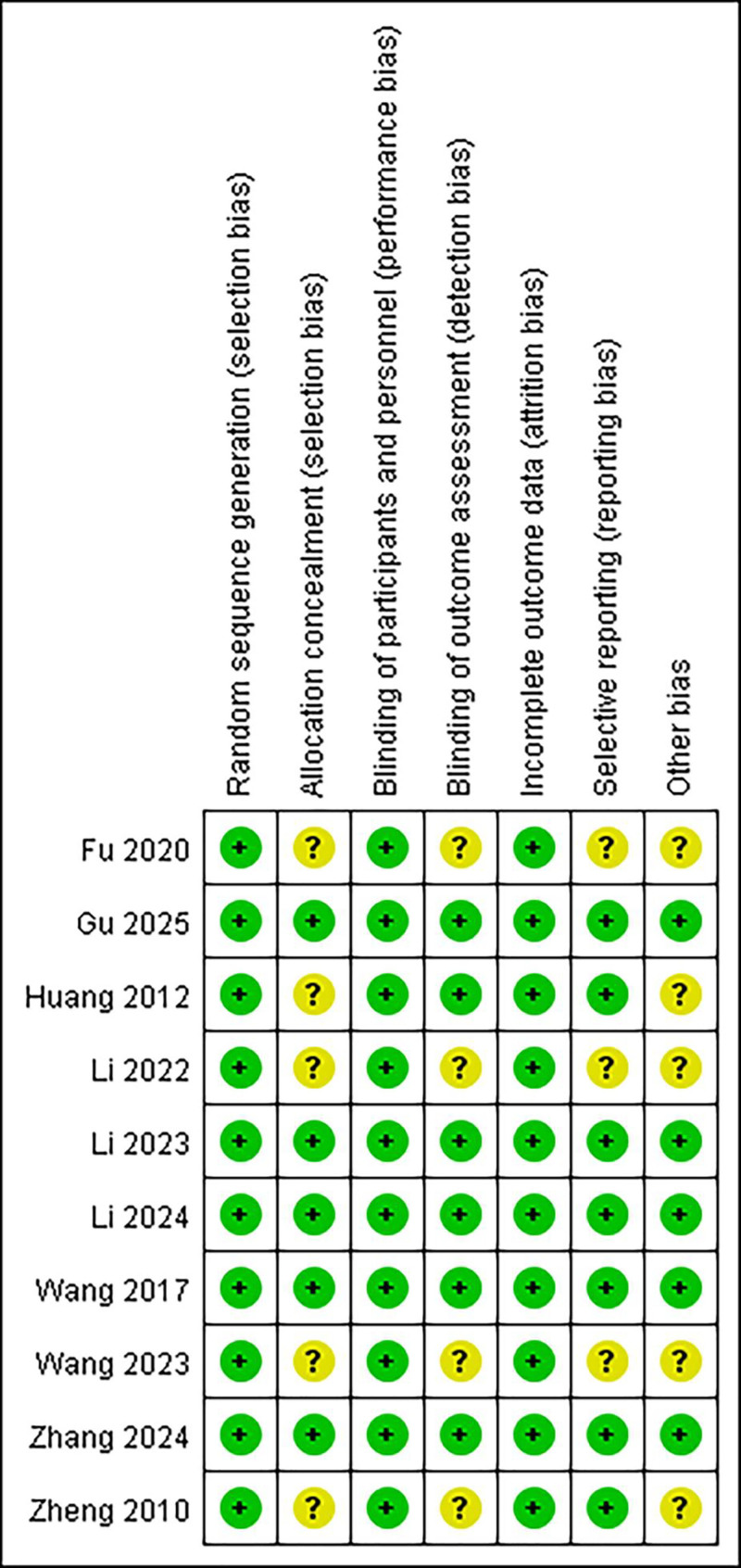
Risk of bias summary for each included study.

**Figure 3.**
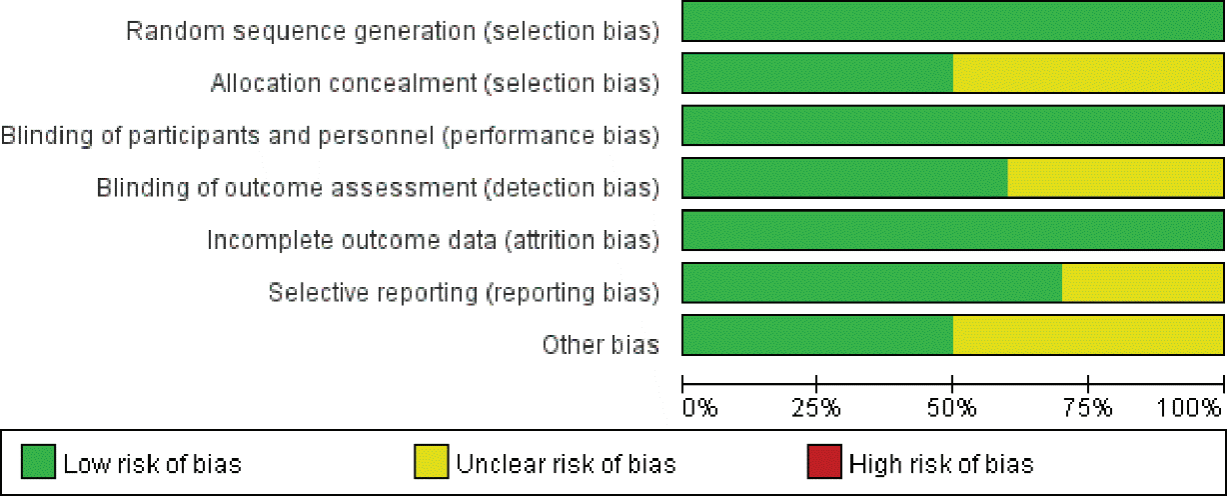
Overall risk of bias across studies by domain.

Risk of bias was assessed using the Cochrane Collaboration’s tool across 7 methodological domains. As shown in Figures 2 and 3, all included studies were rated as low risk for random sequence generation (selection bias) and blinding of participants and personnel (performance bias), indicating robust design in these areas. In the domain of allocation concealment, only 50% of the studies provided sufficient methodological detail and were judged as low risk, while the remaining half were rated as unclear. Similarly, other bias was assessed as low risk in 5 studies and unclear in the remaining 5. For blinding of outcome assessment (detection bias), 6 studies were rated as low risk, whereas 4 were classified as having unclear risk due to lack of sufficient reporting. Regarding selective reporting, 7 studies were considered low risk, and 3 were rated as unclear risk. All studies were assessed as low risk in the domain of incomplete outcome data (attrition bias), suggesting minimal missing data and adequate reporting of follow-up. Taken together, while most domains demonstrated low risk across the included trials, methodological limitations related to allocation concealment, outcome assessor blinding, selective reporting, and other potential biases were present in several studies and should be considered when interpreting the pooled findings.

### 3.3. Outcome measures

#### 3.3.1. HAMD

**Figure 4.**
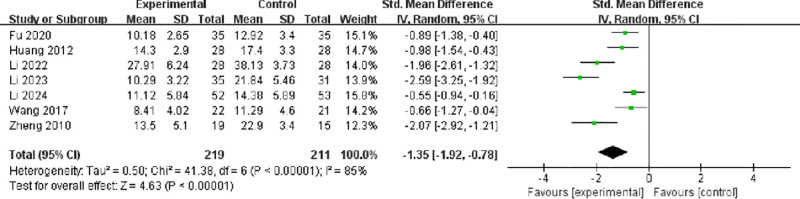
Forest plot of SMDs in HAMD scores between active and sham stimulation. CI = confidence interval; IV = inverse variance; M–H = Mantel–Haenszel; SD = standard deviation; SMD = standardized mean difference.

A total of 7 studies involving 430 participants reported posttreatment HAMD scores.^[9,11,13–15,18,19]^ Significant heterogeneity was observed among the included studies (*I*² = 95%, *P* < .00001); therefore, a random-effects model was applied. The pooled analysis showed that active transcranial magnetic stimulation significantly reduced HAMD scores compared to sham stimulation (SMD = −1.35, 95% CI: −1.92 to −0.78, *P* < .00001; Fig. 4), indicating a strong antidepressant effect.

#### 3.3.2. Response rate

**Figure 5.**
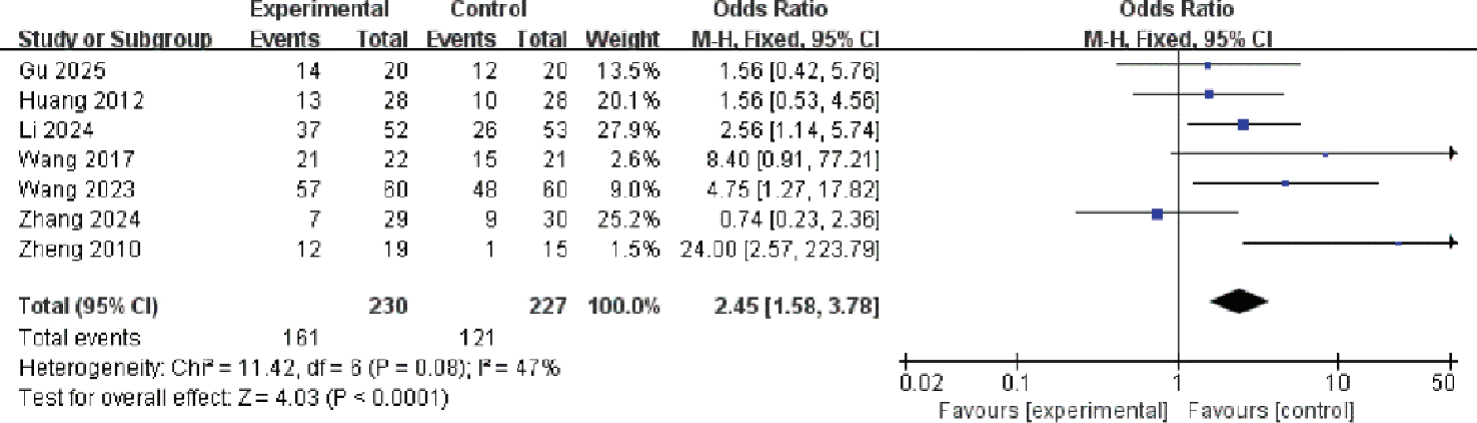
Forest plot of odds ratios for response rate between active and sham stimulation. CI = confidence interval; IV = inverse variance; M–H = Mantel–Haenszel.

Seven studies involving a total of 457 participants reported response rates.^[10,11,13,15–18]^ Moderate heterogeneity was observed among the studies (*I*² = 47%, *P* = .08), and a fixed-effects model was applied. The pooled analysis demonstrated that active transcranial magnetic stimulation significantly improved response rate compared with sham stimulation (odds ratio [OR] = 2.45, 95% CI: 1.58–3.78, *P* = .0001; Fig. 5).

#### 3.3.3. Remission rate

**Figure 6.**
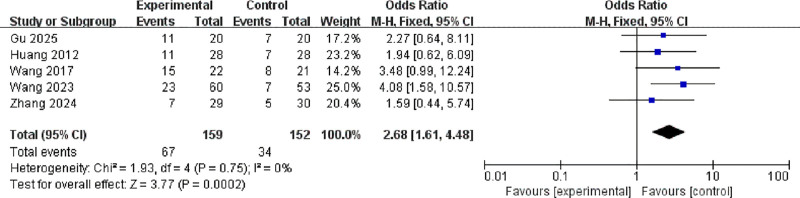
Forest plot of odds ratios for remission rate between active and sham stimulation. CI = confidence interval; IV = inverse variance; M–H = Mantel–Haenszel.

Five studies with a total of 311 participants reported remission rates.^[10,11,15–17]^ No significant heterogeneity was observed (*I*² = 0%, *P* = .75), so a fixed-effects model was applied. Pooled results showed that active transcranial magnetic stimulation significantly improved remission rate compared to sham stimulation (OR = 2.68, 95% CI: 1.61–4.48, *P* = .0007; Fig. 6).

### 3.4. Adverse events

**Figure 7.**
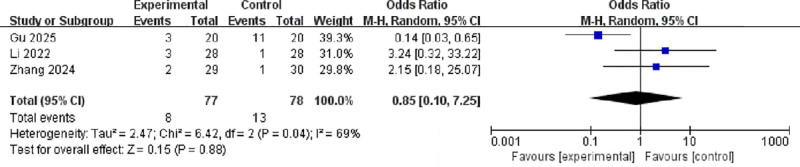
Forest plot of odds ratios for dizziness between active and sham stimulation. CI = confidence interval; IV = inverse variance; M–H = Mantel–Haenszel.

Three studies reported the incidence of dizziness, with a total of 155 participants.^[10,13,17]^ Moderate heterogeneity was observed (*I*² = 69%, *P* = .04), and a random-effects model was applied. The pooled result showed no significant difference between the experimental and control groups (OR = 0.95, 95% CI: 0.12–7.51, *P* = .88; Fig. 7).

**Figure 8.**
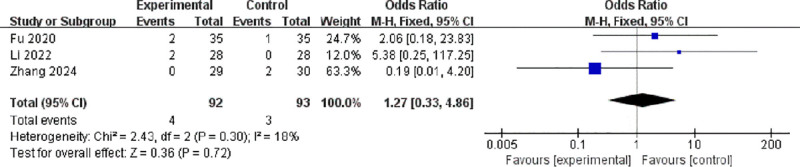
Forest plot of odds ratios for nausea between active and sham stimulation. CI = confidence interval; IV = inverse variance; M–H = Mantel–Haenszel.

Three studies involving 185 participants reported the occurrence of nausea.^[9,14,17]^ No significant heterogeneity was found (*I*² = 18%, *P* = .30), and a fixed-effects model was used. The pooled analysis indicated no significant difference between groups (OR = 1.27, 95% CI: 0.33–4.86, *P* = .72; Fig. 8).

**Figure 9.**
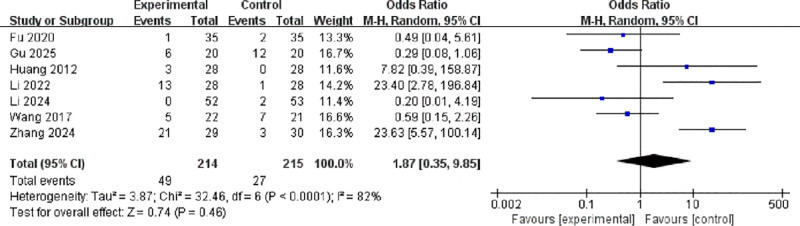
Forest plot of odds ratios for headache between active and sham stimulation. CI = confidence interval; IV = inverse variance; M–H = Mantel–Haenszel.

Seven studies reported headache as an adverse event among 429 participants.^[9–11,13–15,17]^ Significant heterogeneity was observed (*I*² = 82%, *P* = .0001), and a random-effects model was adopted. No statistically significant difference was found in headache incidence between the groups (OR = 1.87, 95% CI: 0.35–9.85, *P* = .46; Fig. 9).

#### 3.4.1. Sensitivity analysis

**Figure 10.**
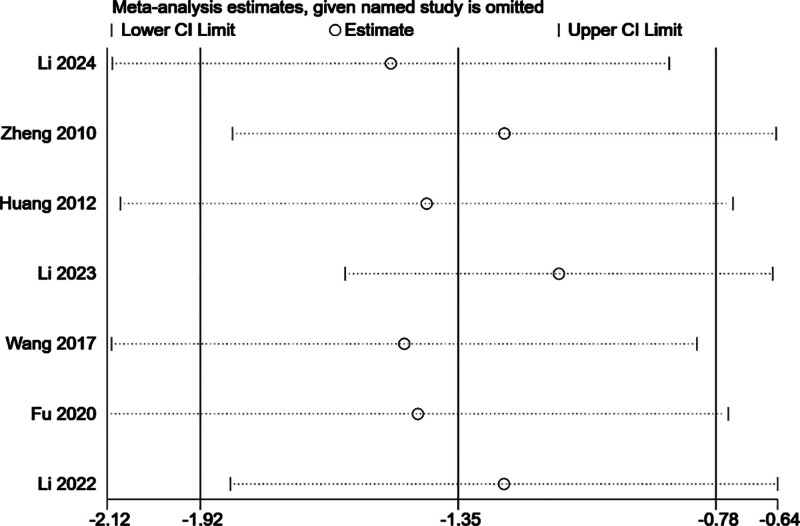
Sensitivity analysis of the effect of active versus sham stimulation on HAMD scores by excluding each study 1 at a time. HAMD = Hamilton Depression Rating Scale.

Sensitivity analysis was performed by sequentially removing each included study to assess the robustness of the pooled effect size for HAMD scores. As shown in Figure 10, the overall results remained consistent regardless of which study was excluded, indicating that no single study significantly influenced the pooled estimate. These findings suggest the robustness and reliability of the meta-analytic results. In addition, a descriptive cross-study comparison based on the study characteristics summarized in Table 1 suggests that the observed heterogeneity in HAMD outcomes may be attributable to differences in intervention duration (ranging from 2–8 weeks) and background antidepressant use (e.g., paroxetine, duloxetine, or none).

#### 3.4.2. Publication bias

**Figure 11.**
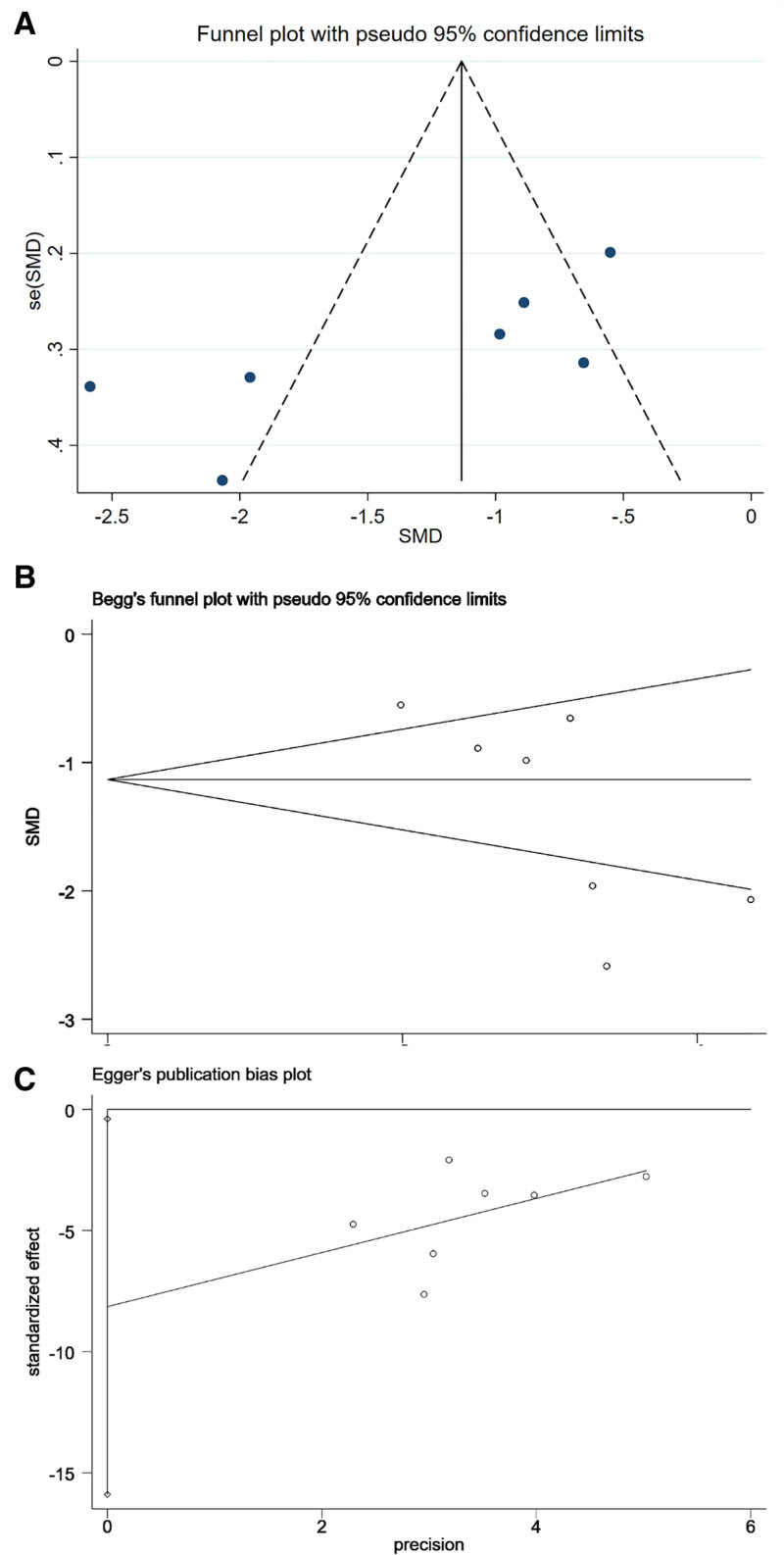
Funnel plot (A) and results of Begg (B) and Egger (C) tests for publication bias.

Publication bias was evaluated using funnel plots along with Begg and Egger tests. As shown in Figure 11A–C, the funnel plot exhibited slight asymmetry. Statistical tests indicated marginal evidence of publication bias, with Begg test (*P* = .051) approaching significance and Egger test (*P* = .043) reaching statistical significance. These results suggest a potential risk of publication bias that should be considered when interpreting the pooled estimates.

#### 3.4.3. Quality of evidence

Applying GRADE to the HAMD reduction across the 7 contributing RCTs, we found a large effect size (SMD − 1.35; 95% CI: −1.92 to −0.78). However, serious inconsistency (*I*² = 95%) and risk-of-bias concerns in several trials led us to rate the overall certainty of evidence as low (Table 2).

**Table 2:** GRADE evidence profile for HAMD‐17 score reduction.

HAMD for MDD.						
Patient or population: patients with MDD. Settings: Intervention: HAMD						
Outcomes	Illustrative comparative risks* (95% CI)		Relative effect (95% CI)	No. of participants (studies)	Quality of the evidence (GRADE)	Comments
	Assumed risk	Corresponding risk				
	Control	HAMD				
HAMD		The mean hamd in the intervention groups was 1.35 standard deviations lower (1.92–0.78 lower)		430 (7 studies)	⊕⊕⊝⊝ Low†	SMD −1.35 (−1.92 to −0.78)

GRADE Working Group grades of evidence.

High quality: further research is very unlikely to change our confidence in the estimate of effect.

Moderate quality: further research is likely to have an important impact on our confidence in the estimate of effect and may change the estimate.

Low quality: further research is very likely to have an important impact on our confidence in the estimate of effect and is likely to change the estimate.

Very low quality: we are very uncertain about the estimate.

CI = confidence interval, GRADE = Grading of Recommendations Assessment, Development and Evaluation (Working Group), HAMD = Hamilton Depression Rating Scale, MDD = Major Depressive Disorder, SMD = standardized mean difference.

* The basis for the assumed risk (e.g., the median control group risk across studies) is provided in footnotes. The corresponding risk (and its 95% confidence interval) is based on the assumed risk in the comparison group and the relative effect of the intervention (and its 95% CI).

† *I*^2^ ≥ 75%.

## 4. Discussion

This systematic review and meta-analysis synthesizes robust evidence from 10 RCTs demonstrating that repetitive transcranial magnetic stimulation, when used adjunctively with selective serotonin reuptake inhibitors (SSRIs), offers significant efficacy and a favorable safety profile in treating major depressive disorder in Chinese patients. The magnitude of therapeutic benefit observed warrants detailed contextualization within the broader literature. The pooled analysis revealed a substantial reduction in Hamilton Depression Rating Scale (HAMD) scores with active stimulation compared to sham control (SMD = −1.35, 95% CI: −1.92 to −0.78; *P* < .00001). This effect size notably exceeds those reported in major international meta-analyses, such as the comprehensive network meta-analysis by Brunoni et al which documented standardized mean differences ranging from −0.55 to −0.89 for repetitive TMS across diverse populations.^[20]^ The significantly enhanced response rate (OR = 2.45, 95% CI: 1.58–3.78) and remission rate (OR = 2.68, 95% CI: 1.61–4.48) further substantiate clinically meaningful outcomes, translating to a number needed to treat of 4 to 5-a favorable metric compared to conventional pharmacotherapies.

Several factors may contribute to the observed superior efficacy within the Chinese population. First, neurobiological differences in treatment response are plausible, as genetic polymorphisms prevalent in East Asian populations (particularly the COMT Val158Met variant influencing prefrontal dopamine metabolism) may potentiate response to neuromodulation.^[21]^ Second, the inclusion of less chronic patients in several Chinese trials contrasts with Western trials predominantly focused on treatment-resistant depression, potentially capturing populations with greater neuroplasticity.^[15]^ Third, the frequent protocol of combining TMS with SSRIs like paroxetine or escitalopram likely yielded synergistic effects, consistent with Chen et al’s meta-analysis demonstrating superior outcomes with combined approaches versus monotherapy.^[22]^ This aligns with mechanistic evidence that TMS enhances monoaminergic neurotransmission and promotes neuroplasticity, potentially overcoming limitations of pharmacotherapy alone.^[23]^

The efficacy extended meaningfully across key demographic subgroups. For adolescents and young adults (a population with rising depression prevalence in China) intermittent theta burst stimulation (iTBS) demonstrated particularly promising remission rates (OR = 2.68).^[10,17]^ This is clinically significant given limited approved treatment options and safety concerns with antidepressants in youth. The maintenance of efficacy in middle-aged and elderly cohorts^[16]^ further supports clinical utility across the lifespan, though age-related neuroanatomical changes may necessitate parameter optimization.^[24]^ While most included studies did not exclusively focus on treatment-resistant depression, the substantial response and remission rates suggest meaningful utility in challenging cases, corroborated by neurobiological findings such as Zheng et al’s observation of prefrontal myo-inositol normalization post-TMS correlating with clinical improvement.^[18]^

The demonstrated effect size (SMD −1.35) holds particular importance when contextualized against first-line antidepressants. As established in Cipriani et al’s landmark network meta-analysis of 21 antidepressant agents, typical effect sizes for SSRIs range between SMD −0.30 to −0.40 in major depression.^[25]^ The magnitude of benefit observed with TMS in this Chinese cohort therefore positions it as a highly effective alternative or augmentative strategy within China’s mental healthcare landscape, especially considering the alarming treatment gap where only 0.5% of MDD patients receive adequate care.^[26]^

The safety profile further supports clinical implementation. No statistically significant differences emerged in dizziness, nausea, or headache incidence between active and sham groups, aligning with international safety databases documenting primarily mild, transient adverse events.^[24]^ The absence of serious adverse events across all included studies reinforces the safety of TMS when applied according to established protocols.

Methodological considerations warrant acknowledgments. The significant heterogeneity (*I*² = 95%) in HAMD outcomes possibly reflects clinical and technical diversity across studies, including variable stimulation parameters (high-frequency rTMS vs iTBS), treatment durations, and concomitant medications. Marginal publication bias detected through Egger test (*P* = .043) suggests potential underrepresentation of smaller negative studies. Limitations in methodological rigor in some trials – particularly regarding allocation concealment and outcome assessor blinding – echo challenges noted in broader TMS literature. In addition, aside from one included RCT conducted with rTMS as a standalone intervention, all other trials administered rTMS adjunctively with antidepressant medication, which limits the feasibility of subgroup analyses to disentangle the independent effects of rTMS from those of combination therapy. Furthermore, future studies should more clearly report and control background antidepressant use – including specific agents, dosages, and treatment durations – and, where feasible, implement balanced monotherapy versus combination therapy arms to enable adequately powered subgroup analyses that disentangle the independent efficacy of rTMS from its synergistic effects with medication. Finally, this review was not prospectively registered in PROSPERO or any other database, which may introduce bias.

This meta-analysis provides compelling evidence that transcranial magnetic stimulation (rTMS/iTBS) demonstrates robust efficacy and favorable safety for Major Depressive Disorder in Chinese patients. The large effect size on core depressive symptoms, coupled with significantly improved response and remission rates, represents clinically meaningful benefits exceeding those typically observed with first-line antidepressants. These findings hold particular significance given China’s substantial mental health treatment gap. Future research should prioritize protocol optimization studies, longitudinal outcome assessments, and validation of predictive biomarkers to guide personalized application across diverse Chinese healthcare settings.

## Acknowledgments

The authors thank the patients for their participation.

## Author contributions

**Conceptualization:** Xianyan He.

**Data curation:** Xianyan He.

**Formal analysis:** Xianyan He.

**Investigation:** Xianyan He.

**Methodology:** Xianyan He.

**Software:** Xianyan He.

**Supervision:** Xianyan He, Xiaofeng Gao.

**Writing – review & editing:** Xiaofeng Gao.
